# Peptide Biomarkers for the Diagnosis of Dengue Infection

**DOI:** 10.3389/fimmu.2022.793882

**Published:** 2022-01-26

**Authors:** Francesca Falconi-Agapito, Karen Kerkhof, Xiomara Merino, Diana Bakokimi, Fiorella Torres, Marjan Van Esbroeck, Michael Talledo, Kevin K. Ariën

**Affiliations:** ^1^ Department of Biomedical Sciences, Unit of Virology, Institute of Tropical Medicine, Antwerp, Belgium; ^2^ Virology Unit, Instituto de Medicina Tropical Alexander von Humboldt, Universidad Peruana Cayetano Heredia, Lima, Peru; ^3^ Hospital Santa Gema, Yurimaguas, Peru; ^4^ Department of Clinical Sciences, National Reference Center for Arboviruses, Institute of Tropical Medicine, Antwerp, Belgium; ^5^ Department of Biomedical Sciences, University of Antwerp, Antwerp, Belgium

**Keywords:** immunoassay, luminex, dengue peptide, arbovirus, seromarkers, random forest, ROC analysis

## Abstract

In a world with an increasing population at risk of exposure to arthropod-borne flaviviruses, access to timely and accurate diagnostic tests would impact profoundly on the management of cases. Twenty peptides previously identified using a flavivirus proteome-wide microarray were evaluated to determine their discriminatory potential to detect dengue virus (DENV) infection. This included nine peptides recognized by IgM antibodies (PM peptides) and 11 peptides recognized by IgG antibodies (PG peptides). A bead-based multiplex peptide immunoassay (MPIA) using the Luminex technology was set-up to determine Ab binding levels to each of these peptides in a panel of 323 carefully selected human serum samples. Sera are derived from individuals either infected with different viruses, namely, the four DENV serotypes, Zika virus (ZIKV), yellow fever virus (YFV), chikungunya virus (CHIKV), West Nile virus (WNV) and Human immunodeficiency virus (HIV), or receiving vaccination against YFV, tick-borne encephalitis (TBEV), and Japanese encephalitis virus (JEV). Additionally, a set of healthy controls were included. We targeted a minimum specificity of 80% for all the analysis. The PG-9 peptide had the best sensitivity (73%) when testing DENV sera from acute patients (A-DENV; <8 days since symptom onset). With sera from convalescent DENV patients (C-DENV; >10 days since symptom onset) the FPG-1 peptide was the best seromarker with a sensitivity of 86%. When combining all A-DENV and C-DENV samples, peptides PM-22 and FPG-1 had the best-diagnostic performance with a sensitivity of 60 and 61.1%, and areas under the curve (AUC) of 0.7865 and 0.8131, respectively. A Random forest (RF) algorithm was used to select the best combination of peptides to classify DENV infection at a targeted specificity >80%. The best RF model for PM peptides that included A-DENV and C-DENV samples, reached a sensitivity of 72.3%, while for PG peptides, the best RF models for A-DENV only, C-DENV only and A-DENV + C-DENV reached a sensitivity of 88.9%, 89.1%, and 88.3%, respectively. In conclusion, the combination of multiple peptides constitutes a founding set of seromarkers for the discrimination of DENV infected individuals from other flavivirus infections.

## 1 Introduction

The World Health Organization (WHO) reported an increase from 3.2 million dengue symptomatic infections in 2015 to 5.2 million in 2019 (1). These numbers however, do not reflect the actual burden of the dengue virus (DENV), because in 2013 a report estimated that the number of dengue infections could reach 390 million annually worldwide (2). The difficulties to spot-on true dengue numbers are mainly attributed to the failure in the surveillance systems unable to capture cases that do not seek healthcare (under-ascertainment); and to report cases that do seek healthcare (underreporting). Among the underreported infections, the under-diagnosis is of specific concern in resource-limited setting (RLS) where insufficient testing, poor deployment of diagnostic tools, and misdiagnosis with other febrile infectious diseases take place. In the Americas in 2020, the vast majority of the North American dengue cases reported to the Pan American Health Organization (PAHO) were lab confirmed, whereas in the Andean sub-region only 25% of the reported dengue cases were confirmed by a lab test (3).

Molecular techniques are preferred for the diagnosis of DENV because of their high sensitivity and specificity, however they are not the most widely applied due to the constraints to deploy them in RLS and the mostly short viremic window during which viral RNA can be detected in the blood. Serological tests on the other hand are more suitable for identifying infected individuals in RLS and tackle the problem of the narrow diagnostic window because anti-DENV antibodies (Abs) remain in the serum for much longer periods. The main concern with Ab-based detection techniques is cross-reactivity by Abs towards antigens (Ags) of other antigenically related flaviviruses, of which proteins can share approximately 60% or higher amino acid sequence identity (4). The detrimental implications of cross-reactivity on the accuracy of the serological tests leads to false-positive test results (5, 6).

The current increasing incidence of epidemics and spread across the tropical and subtropical world of different flaviviruses (7, 8) and immunization against different flaviviruses with vaccines for yellow fever virus (YFV), tick-borne encephalitis virus (TBEV), and Japanese encephalitis virus (JEV) (9, 10), reinforce the importance importance for developing high-quality serological tests not only to accurately identify DENV infections, but also in distinguishing past from current infections to determine serostatus for DENV pre-vaccination screening (11, 12). The accurate discrimination of anti-DENV Abs from Abs raised against related flaviviruses, with tests that can be properly deployed to LRS providing same-day results would significant impact on (i) offering opportune clinical management of DENV cases, (ii) determining suitability for vaccination, and (iii) improving surveillance systems for a better support of control intervention programs based on more reliable evidence-based decision making.

DENV serological diagnosis includes a wide spectrum of different formats and Ag designs (13), however low specificity has constantly been reported for these tests due to the presence of impurities in Ags widely used in commercial tests such as whole viral lysates. Recombinant proteins such as NS1 and Envelope proteins have helped in reducing false positivity and were rapidly adapted to different formats such as indirect ELISA, MAC-ELISA, and lateral flow devices for their commercial application (4, 14–17). Nevertheless, despite the progress made with the operational characteristics and good sensitivity, challenges with false positivity remain especially with the increasing co-circulation of arthropod-borne flaviviruses across the globe (4, 18–21). Therefore, more appropriate biomaterials that involves the selection of fragments with low sequence identity to proteins other than target are needed to circumvent the issues of cross-reactivity.

In previous work, we screened a 15-mer peptide microarray library covering the entire proteomes of the four DENV serotypes, ZIKV and YFV and identified 20 immunodominant peptides that were recognized by the sera of DENV infected individuals (22). Using a Using a bead-based multiplex peptide immunoassay (MPIA), we report here the diagnostic potential of the selected synthetic peptides on a larger panel of carefully selected sera from individuals previously infected with DENV, ZIKV, YFV, WNV, CHIKV, and HIV or receiving vaccination against YFV, TBEV or JEV. This study validates our initial findings with respect to the specificity of the selected peptides for detecting anti-DENV Abs in clinical specimens and offers strong supportive evidence for the application of specific peptide combinations for next-generation DENV diagnostic tests.

## 2 Materials and Methods

### 2.1 Human Serum Samples

#### 2.1.1 Endemic Samples

From a prospective longitudinal study carried out between July 2018 and March 2019 in the Santa Gema Hospital (SGH) in Yurimaguas, Peru, 136 patients with acute undifferentiated febrile illness, with a temperature ≥37°C for 7 days or less, together with at least one of the following symptoms: arthralgia, myalgia, head ache or rash, aged between 5 and 65 years old, were enrolled regardless of gender and ethnicity. DENV infection was confirmed by RT-PCR (n= 49 patients), as previously described (23). Four additional patients recruited in Iquitos, Peru in April 2018, positive for DENV by RT-PCR were also included. Samples were subsequently serotyped as DENV-2 using a multiplex RT-PCR protocol previously reported (24, 25). A subset of 32 DENV patients were followed up and serum samples were collected up to 217 days after symptom onset (DASO). During this time, one, two or three additional serum samples were obtained making a total of 119 samples. Follow-up samples were collected depending on the willingness of the patient to donate additional samples for the study.

Two serum samples from Peruvian individuals with a YFV infection collected in 2007 were included in the set of DENV-negative samples. YFV infection was confirmed by clinical symptomatology and the presence of IgM Abs by using an in-house IgM capture ELISA developed by the National Institute of Health from Peru. Both patients had not received yellow fever vaccination at the time the samples were collected.

#### 2.1.2 Non-Endemic Samples

Biobanked samples from travelers consulting the travel clinic at the Institute of Tropical Medicine Antwerp, Belgium, hosting the national reference center for arboviruses were selected. Returning travelers with a recent DENV infection (n = 18) confirmed by RT-PCR or IgM and/or IgG detection were included. A panel of 65 serum samples from Belgian citizens receiving vaccination against the flaviviruses TBEV (n = 16), JEV (n = 10), and YFV (n = 22) were included in the analysis. For YFV, follow-up serum samples were obtained from some individuals up to 1 year after receiving vaccination, making a total of 39 serum samples. Belgian travelers returning from arbovirus endemic areas with a RT-PCR or a serology positive test for ZIKV (n = 58), WNV (n = 8) or CHIKV (n = 18) infection were also included. A set of 18 serum samples from HIV infected individuals were included as controls (**Table 1**).

**Table 1:** Overview of samples used in the study.

Place	Virus	Infection/vaccination	Number of individuals	Number of samples	Age, years	Confirmatory test
Yurimaguas, Peru	DENV	Infection	49	110	6–64	RT-PCR (23)
Iquitos, Peru	DENV	Infection	4	9	8–65	RT-PCR (23)
Peru	YFV	Infection	2	2	>18	In-house capture IgM ELISA (NIH, Peru)
Lima, Peru	Healthy donor		1	1	>18	N.A.
Institute of Tropical Medicine (Antwerp, Belgium)	DENV	Infection	18	18	>18	RT-PCR/ELISA (IgM-IgG) (26)
	ZIKV	Infection	58	58	>18	RT-PCR/ELISA (IgM-IgG)/IFA (IgM-IgG)/In-house VNT (27)
	YFV	Vaccination	22	39	>18	In-house PRNT90 adapted from (28); Anti-Yellow fever IIFT (IgM/IgG)—Euroimmun
	TBEV	Vaccination	16	16	>18	In-house VNT90
	JEV	Vaccination	10	10	>18	In-house VNT90
	WNV	Infection	8	8	>18	RT-PCR/ELISA (IgM) Capture DxSelectTM/ELISA IgG DxSelectTM; Focus Diagnostics (29)
	CHIKV	Infection	18	18	>18	RT-PCR/IFA (IgM-IgG) Euroimmun (29)
	HIV	Infection	18	18	>18	Enzygnost Anti-HIV 1/2 Plus Vironostika HIV Uniform II Ag/Ab; Innotest HIV Ag mAb; InnoLIA HIV I/II Score
	Healthy donors		16	16	>18	N.A.

DENV, dengue virus; YFV, yellow fever virus; ZIKV, Zika virus; TBEV, tick-borne encephalitis virus; JEV, Japanese encephalitis virus;

WNV, West Nile virus; CHIKV, chikungunya virus; HIV, Human immunodeficiency virus;

RT-PCR, real time PCR; VNT90, virus neutralization test; PRNT90, plaque reduction neutralization test;

IFA, immunofluorescence assay; IIFT, indirect immunofluorescence test; NA, not applicable.

#### 2.1.3 Negative Samples From Healthy Blood Donors

Sixteen samples from healthy citizens of the city of Antwerp were selected from a panel of sera with ethical approval for broad Ab testing. One serum sample from a citizen of the city of Lima with no register of visiting endemic areas or receiving the yellow fever vaccine, who tested negative to flavivirus IFAT (Euroimmun, Lübeck, Germany) was included in the analysis (**Table 1**).

For the assessment of the potential use of the peptides for the diagnosis of DENV, we considered as positive samples those sera from patients with a DENV infection confirmed by RT-PCR (endemic individuals), and/or by IgM or IgG detection (returning travelers). The negative samples included sera from individuals with history of flavivirus exposure either by infection or vaccination, sera from CHIKV patients, sera from HIV patients, and sera from healthy donors. The presence of anti-DENV antibodies was not ruled out in the negative sample set.

All serum samples were heat inactivated at 56°C for 30 min before serological analyses.

### 2.2 Peptides

**Figure 1:**
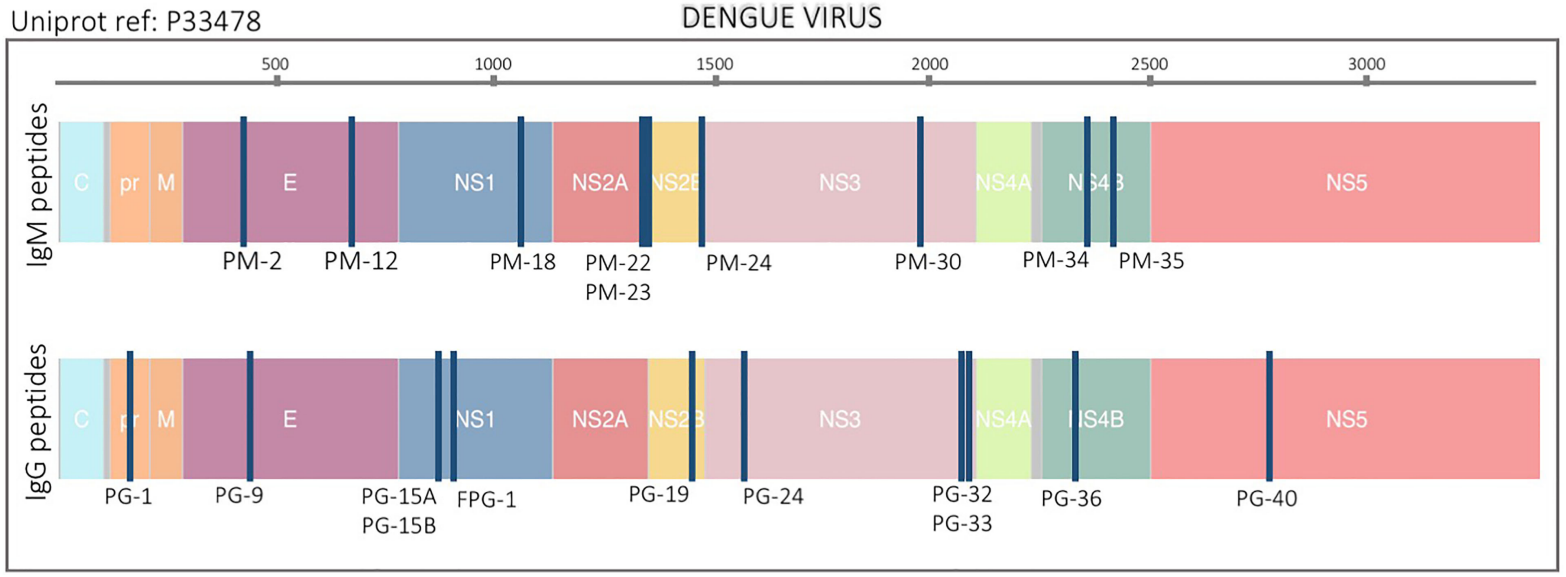
Location of peptide biomarkers in the DENV proteome. Selected peptides are plotted relative to the polyprotein coordinates (start amino acid position) for DENV (P33478). Each blue vertical bar represents a single peptide. Top and bottom plots are IgM and IgG peptides respectively. Structural proteins: Capsid (C), Membrane (pr, M) and Envelope (E), and non-structural proteins: NS1, NS2A, NS2B, NS3, NS4A, NS4B, and NS5 are color coded. The peptide names are also indicated.

Nine peptides recognized by IgM Abs (PM peptides) located in the E, NS1, NS2a, NS2b, NS3, and NS4b proteins, and 11 peptides recognized by IgG Abs (PG peptides) located in the prM, E, NS1, NS2b, NS3, NS4b, and NS5 proteins and that were previously identified to be highly immunogenic (22) were selected for further analysis. **Figure 1** shows the location of the peptides in the DENV proteome. Peptide synthesis was done according to standard protocol by using solid-phase 9-fluorenylmethoxycarbonyl (Fmoc) chemistry with automated synthesizers (Genecust, Boynes, France). A cysteine was added to the N-terminus to facilitate conjugation of BSA. The purity of peptides was confirmed to be >95% by Mass spectrum analysis and HPLC. Peptide information is detailed in **Table 2**. Additionally, the viral lysates (VL) from the four DENV serotypes (D1–4) (ZeptoMetrix^®^, NY, USA) were included as positive controls in the analysis.

**Table 2:** Overview of the peptides used in the study.

Antibody class	Peptide	Sequence (N-terminal to C-terminal)	g/mol	Isolelectric point	Length	DENV serotype	DENV protein	Polyprotein position	
								Start	End
IgM	PM-2	CVTKLEGKIVQYENL	1,737.03	5.71	15	DENV1	E	401	415
	PM-12	CPPFGESNIVIGIGDK	1,645.88	4.18	16	DENV3	E	654	668
	PM-18	CAGPWHLGRLEMDFDF	1,894.15	4.36	16	DENV2	NS1	1,039	1,053
	PM-22	CPMAVAAMGVPPLPLF	1,614.06	5.28	16	DENV3, DENV4	NS2A	1,319	1,333
	PM-23	CTAIFLTTLSRTSKKR	1,826.18	11.65	16	DENV2	NS2A	1,330	1,344
	PM-24	CVFPVSIPITAAAWYL	1,751.1	5.28	16	DENV2	NS2B	1,453	1,467
	PM-30	CEPLENDEDCAHWKEA	1,888.99	3.84	16	DENV2, DENV3	NS3	1,951	1,965
	PM-34	CLLAIGCYSQVNPITL	1,708.06	5.28	16	DENV2	NS4B	2,336	2,350
	PM-35	CAIDLDPVVYDAKFEK	1,826.08	4.06	16	DENV1	NS4B	2,396	2,410
IgG	PG-1	CDGVNMCTLMAMDLGE	1,703.02	3.38	16	DENV2	prM	143	157
	PG-9	CENLKYTVIITVHTGD	1,806.05	5.39	16	DENV3	E	413	427
	PG-15A	CNELNYILWENNIKLT	1,980.25	4.26	16	DENV3, DENV4	NS1	847	861
	PG-15B	CNELNYVLWEGGHDLT	1,863.02	3.93	16	DENV4	NS1	847	861
	FPG-1	CMELKYSWKTWGKAKI	1,972.39	10.02	16	DENV3, DENV4	NS1	883	897
	PG-19	CEEEEQTLTILIRTGL	1,848.09	3.82	16	DENV2, DENV3	NS2B	1,433	1,447
	PG-24	CISYGGGWRFQGSWNT	1,818.97	7.74	16	DENV1, DENV2	NS3	1,551	1,565
	PG-32	CKEGERKKLRPRWLDAR	2,141.51	11.02	17	DENV1, DENV2	NS3	2,059	2,073
	PG-33	CRKKLRPRWLDARTYSD	2,164.5	10.86	17	DENV1, DENV2	NS3	2,063	2,077
	PG-36	CANQAVVLMGLDKGWP	1,702.02	5.55	26	DENV1	NS4B	2,312	2,326
	PG-40	CTRHVAVEPEVANLDI	1,765.99	4.42	16	DENV1	NS5	2,753	2,767

### 2.3 Multiplex Peptide Immunoassay (MPIA)

We used a serological assay based on the Luminex technique to measure IgM and IgG immunoglobulin levels. The covalent coupling of the nine IgM peptides, eleven IgG peptides and the four VL-D1-4 Ags to paramagnetic MagPlex 6.5 μm COOH-microspheres from Luminex Corporation (Austin, TX) was carried out as previously described (30, 31). In brief, all peptides and the four VLs were coupled at a concentration of 5 μg/ml for 10^6^ beads/μl. IgG and IgM Abs were measured in separate assays. A mixture of the antigen-coupled microspheres was prepared in a hypertonic phosphate buffered saline, 1% BSA, 0.05% Sodium Azide solution (PBS-BN) to a final concentration of 1,000 beads/antigen/well. A mixture of 25 μl of coupled microspheres was added to a 96-well μClear^®^ flat bottom microplate (Falcon™ 353072). Serum diluted 1:100 was found to be the optimal serum concentration to measure both IgM and IgG Abs based on serial dilution standardization. For the detection of IgM Abs, GullSORB™ IgG inactivation reagent (Meridian Bioscience™) in 1:10 dilution was added to the sera to remove the rheumatoid factor prior to testing. The wells were then incubated with 50 μl of human serum diluted 1:100 in blocking solution (PBS-TBN: PBS-BN + 0.05% Tween). Microplates were incubated for 1 h at room temperature (RT) on a plate shaker (600 rpm) in the dark. The plates were washed three times with PBS-TBN. Following the washing step, the PE-conjugated secondary goat anti-human IgG or anti-human μ-chain IgM (Jackson Immuno Research, PA, USA) Ab diluted 1:125 in 100 μl PBS-TBN per well was added for 45 min at RT on a plate shaker (600 rpm) in the dark. After a second washing step, beads were resuspended in 150 μl of PBS-BN. Plates were put on the plate shaker (900 rpm) in the dark for 5 min before reading. Data was acquired using a Luminex^®^ 100/200 analyzer. Results were expressed as median fluorescent intensities (MFI). All tests were performed in duplicates and in two independent experiments. Raw data with the MFI for each of the peptides is detailed in **Supplementary Table 1**.

### 2.4 Diagnostic Performance of the DENV Peptides

The Receiver operating characteristic (ROC) curves, their corresponding area under the curve (AUC), and the specificity and sensitivity of the IgM and IgG assays, were established using all 137 DENV-positive samples (acute and convalescent), and 186 DENV-negative samples (ZIKV, TBEV, JEV, WNV, CHIKV, HIV, and negative control sera). Acute samples from endemic patients were also included for the classification performance because most of these were IgG positive in DENV-ELISA probably as a result of previous flavivirus infections. The serum samples coming from DENV positive cases were stratified into two groups based on the time since onset of symptoms: (i) acute samples (A-DENV), ≤8 DASO, N = 54 and (ii) early convalescent samples (E-DENV), ≥10 and ≤70 DASO, N = 46. A third group (iii) was made that contains all samples: A-DENV, E-DENV and late convalescent (L-DENV), ≥71 up to 230 DASO, N = 38. The trade-off between sensitivity and specificity was graphically displayed with the ROC curve analyses. Based on this analysis cut-off values were assigned to each peptide in single-plex. We calculated the AUC and selected three targets: a sensitivity of at least 80%, and with this constraint, (i) the specificity is maximized, ii) the sensitivity and specificity are equally weighted, so that a combination that maximizes sensitivity and specificity is selected, and iii) when a specificity of at least 80% is enforced, the sensitivity is maximized.

To assess the prediction capacity of peptide combinations, ROC curves, their correspondent AUC, and the specificity and sensitivity of the IgM and IgG assays were calculated using the predicted values estimated by supervised machine learning Random Forest (RF) algorithm models as implemented in the R-package ‘randomForest’ (32). Samples were stratified and randomly spliced into a training and a test set. The training samples were used to fit a random forest classifier which then predicted the negative vs. positive category of unseen test samples (2/3 of total samples). This was repeated n = 50,000 times. Variable (each antigen) importance was assessed using the ‘varImplot’ function of the same package and was ranked according to the ‘mean decrease in accuracy’ and ‘mean decrease in Gini’. The peptides ranking in the top six of the ‘mean decrease in accuracy’ were selected for the training and cross-validation analysis. Three RF models were built for PM peptides (RFM) and PG peptides (RFG), namely, (i) A-DENV samples (RFM1 and RFG1), (ii) E-DENV samples (RFM2 and RFG2), and (iii) A-DENV+E-DENV+L-DENV (RFM3 and RFG3). Each of these positive control sets was analyzed with the negative samples (n = 185). For each model, we calculated the AUC and enforced a specificity target of at least 80%, and based on these constraints, sensitivity was maximized. Classification algorithms implemented in R (version 3.6.3) were adapted from Rosado et al. (33).

The R Stats package was used to perform this analysis and Spearman’s rho statistics was used to estimate the rank-based measure of association. Heatmaps based on the calculated rho-scores were created for the analyzed IgM and IgG peptides. For all analyses, differences with probabilities of p <0.05 were considered statistically significant. Differences in measured Ab responses were assessed using the non-parametric (Steel-Dwass) tests for independent pair-wise comparison with Bonferroni adjustment. Differences in classification performance were assessed by pairwise comparison using McNemar’s test.

### 2.5 Ethical Clearance

The study was approved by the ethical review boards of the Peruvian University Cayetano Heredia, Peru (Protocol No. 101480), the Institute of Tropical Medicine Antwerp, Belgium (Protocol No. ITG 1304/19) and the University of Antwerp, Belgium (Protocol No. 19/42/477). This study was conducted in compliance with the ethical standards of the latest amended Declaration of Helsinki and of the International Conference Harmonization (ICH) guidelines, plus adhering to local laws and regulations.

## 3 Results

### 3.1 Antibody Levels Against DENV Peptides

In a previous study, using a high throughput 15-mer peptide microarray, immunogenic epitopes recognized by IgM and IgG Abs present in the sera of confirmed DENV infected individuals were identified. In order to further evaluate their diagnostic capacity, two multi-antigen assays were developed, one with the nine PM peptides recognized by IgM Abs and a second with ten PG peptides out of the eleven peptides recognized by IgG Abs. The PG-36 peptide was unable to couple to the microspheres and therefore was not included in the multiplexing. For each assay, the peptides plus the VLs (containing the entire viral proteome) of the four DENV serotypes were immobilized on microspheres to allow the detection of IgM and IgG Abs in a panel of 323 serum samples of DENV, ZIKV, YFV, TBEV, JEV, WNV, CHIKV, HIV, and negative healthy controls.

When assessing the levels of IgM and IgG Abs directed towards the peptides, the MFI values for most of the peptides were significantly higher in DENV-infected individuals compared to the levels in the DENV-negative samples that included sera from individuals with exposure to other flavivirus (**Supplementary Table 2**; Positive vs Negative, *P* <.05), however in four out of the 19 peptides evaluated (i.e., PG-9, PG-15A, PG-19, and PG-24) this difference was not significant (*P* >.05).

**Figure 2:**
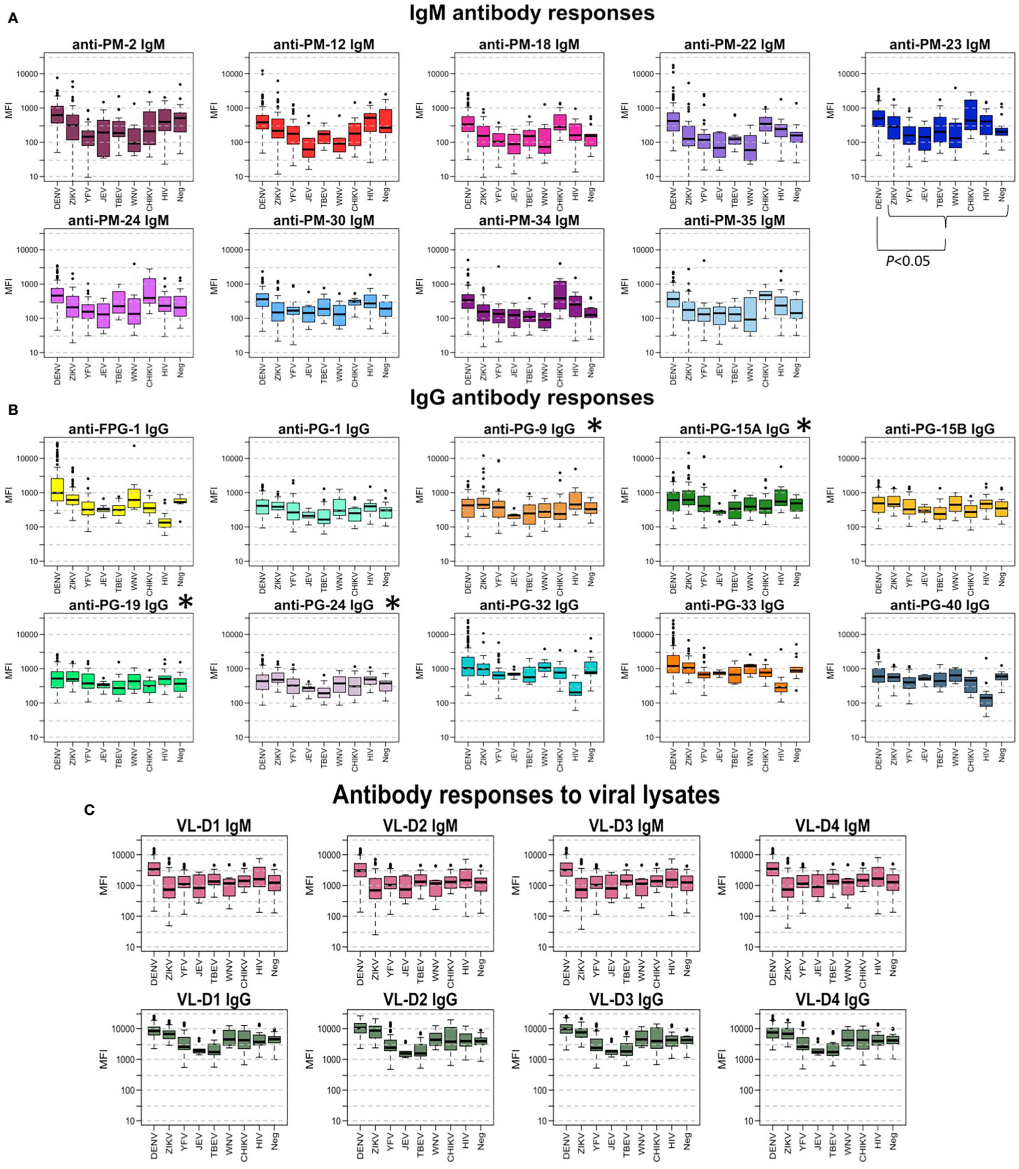
Antibody responses to peptide biomarkers. **(A)** Boxplot display of the IgM response against the peptides in median fluorescence intensity (MFI) values in serum samples for each virus group. Positive DENV samples (n = 137) where confirmed by RT-PCR in endemic patients and RT-PCR and/or IgM/IgG seroconversion in travelers. DENV-negative samples (n = 185) corresponded to ZIKV, YFV, TBEV, JEV, WNV, CHIKV, HIV, and negative controls from non-endemic healthy donors. Horizontal box boundaries and midline denote sample quartiles. **(B)** Boxplot display of the measured IgG against the biomarkers in MFI values in serum samples from each group. **(C)** Boxplot display of the measured IgM and IgG (MFI) against the viral lysates (entire proteome) of the four DENV serotypes in serum samples from each group. Significant differences (P<.05) between MFI values in DENV-infected individuals compared to the response from people with exposure to other flaviviruses was found for all peptides, except for those labeled with *.

**Figure 2** shows the comparison between the Ab response against each peptide for the different groups of samples. In general, the measured IgG levels against the evaluated Ags were higher than the IgM levels. From the graph, it can be seen that IgM levels against PM-22 and IgG levels against FPG-1 were clearly higher in the DENV group than in the other groups of samples. Sera from individuals with a flavivirus history of infection (ZIKV, WNV) or vaccination (YFV, TBEV, JEV), showed lower IgM titers against PM peptides, but similar IgG levels against the PG peptides when compared with the Ab levels from DENV infected individuals. Of note, high IgM Ab levels against peptides PM-18, PM-22, PM-23, PM-24, PM-34, and PM-35 were observed in CHIKV infected individuals.

When analyzing only the dengue positive samples, we stratified the samples into different groups in an attempt to determine if the biomarkers could differentiate between: (i) endemic vs. non-endemic samples, (ii) hospitalized vs. non-hospitalized, and (iii) acute vs. convalescent samples (**Supplementary Table 2**). The Ab levels against peptides PM-2, PM-35, PG-1, PG-15B, PG-19, and PG-40 were significantly higher in samples coming from endemic individuals in comparison to the Ab levels measured in non-endemic samples (*P* <.05). DENV patients that were hospitalized after presenting severe symptoms of DENV showed significantly higher IgG levels against PG-33 compared to the Ab levels in non-hospitalized patients (*P* = .0087). The IgG levels against FPG-1 were higher in E-DENV compared to A-DENV samples (*P* <.001) and to L-DENV (*P* <.05) (**Supplementary Figure 1**).

Next, we represented the Ab-binding data of the PM and PG peptides using a cell plot. The FPG-1 peptide showed a clear discriminatory response, higher titers (red cells) are observed in DENV positive samples, while sera from individuals with exposure to arboviruses other than DENV show low MFI values against this peptide (blue cells) (**Supplementary Figure 2**).

### 3.2 Performance Assessment of the DENV Peptide Biomarkers

**Figure 3:**
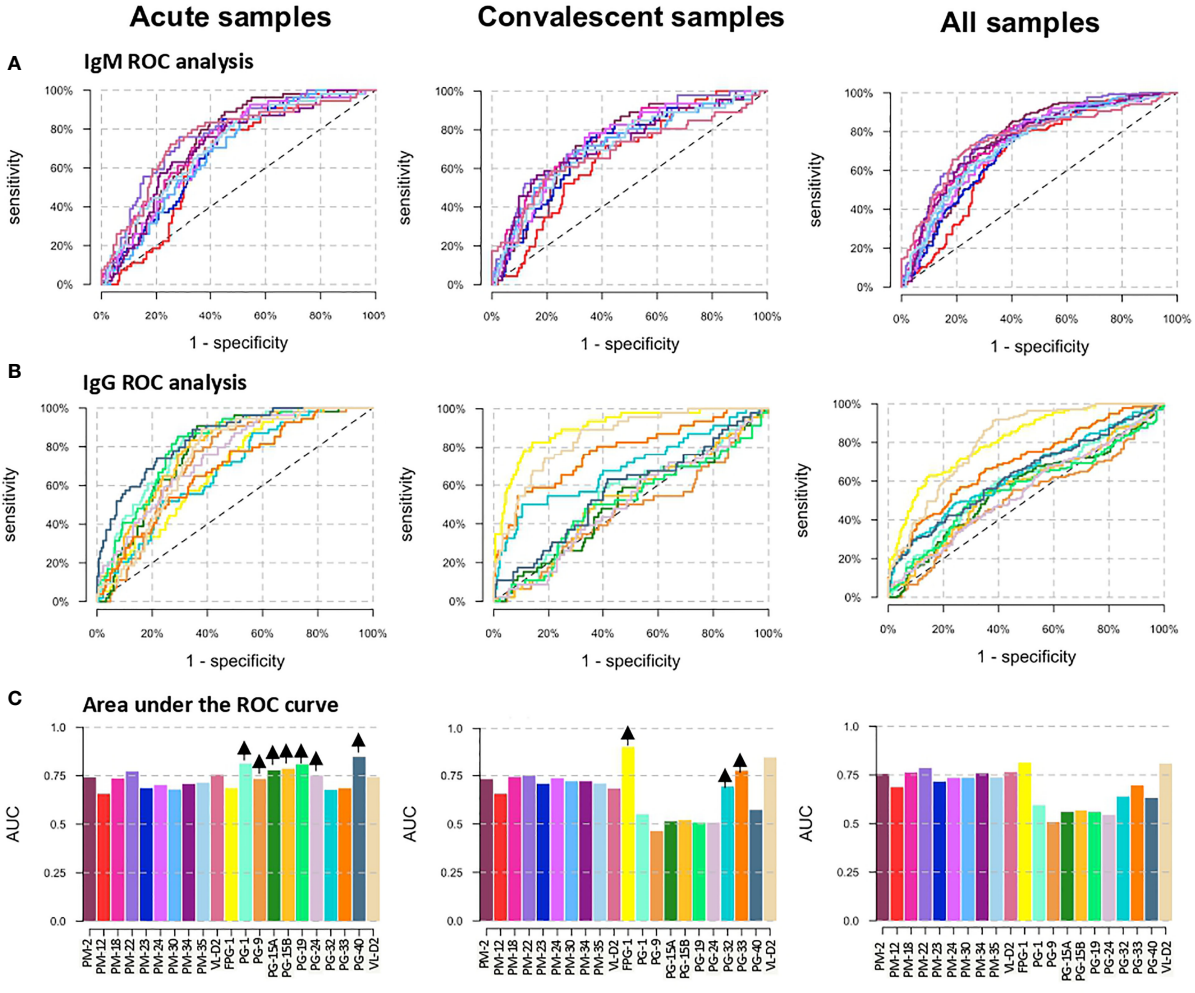
Diagnostic performance of the peptide biomarkers. **(A)** ROC curves for IgM peptides and **(B)** IgG peptides. The three columns correspond to the separate analysis of the data considering as positive DENV samples: acute samples only (left graphs), convalescent samples only (center graphs) and acute + convalescent (right graphs). Colors correspond to Abs against the different antigens as shown in panel **(C)**. **(C)** Area under the ROC curve (AUC) for individual biomarkers. Arrows in the top of each bar indicates an increase in the AUC of the peptide when only acute DENV or convalescent DENV samples are included as positive controls in the analysis.

ROC curves were used to compare the diagnostic value of the 19 peptides individually (**Figure 3**). High AUCs mean high specificity and high sensitivity and, therefore, a greater predictive capacity of the test. For the PM peptides, when DENV positive samples were analyzed separately into acute and convalescent samples, their diagnostic performance in terms of AUCs were lower compared to the analysis of all samples (acute and convalescent) together (**Figures 3A, C** and **Supplementary Table 3**). On the other hand, when only acute samples were included in the analysis, the AUC of peptides PG-1, PG9, PG15A, PG15B, PG-19, and PG-24 increased, while if only convalescent samples were included, FPG1, PG-32, and PG-33 showed an improvement in their diagnostic performance (**Figures 3B, C**). When specificity was targeted to at least 80%, PM-22 and FPG-1 were the best biomarkers to diagnose DENV infections regardless of the time after symptom onset, with specificity, sensitivity and AUC values of 80.4%, 60.3%, and 0.7865 for PM-22 and 80.5%, 61.1%, and 0.8131 for FPG-1, respectively (**Supplementary Table 3**). When only A-DENV samples were included in the analysis, the best PG biomarkers to detect DENV infection are PG-1, PG-19, and PG-40 with 69.7%, and 0.8114, 69.7%, and 0.8114, and 69.7%, and 0.8114, of sensitivity and AUC respectively and minimally 80% specificity. The diagnostic performance of FPG-1 improved when only E-DENV samples were included in the analysis, reaching a sensitivity of 86.5% and an AUC of 0.9031 (**Supplementary Table 3**). For a targeted 80% specificity, cut-off values were calculated and the classification outcomes (positive or negative) were obtained for each peptide. Based on this classification, discriminatory test performance was performed for the peptides using the McNemar’s and Cohen’s kappa tests. When the classification outcome of each peptide was pairwise compared with the reference classification (DENV-positive and DENV-negative, **Table 1**), FPG-1 and PM-22 rendered the best discriminatory test performance (McNemar’s test: **Supplementary Table 4**) and the highest agreement with respect to the reference (Cohen’s kappa test: **Supplementary Table 5**).

**Figure 4:**
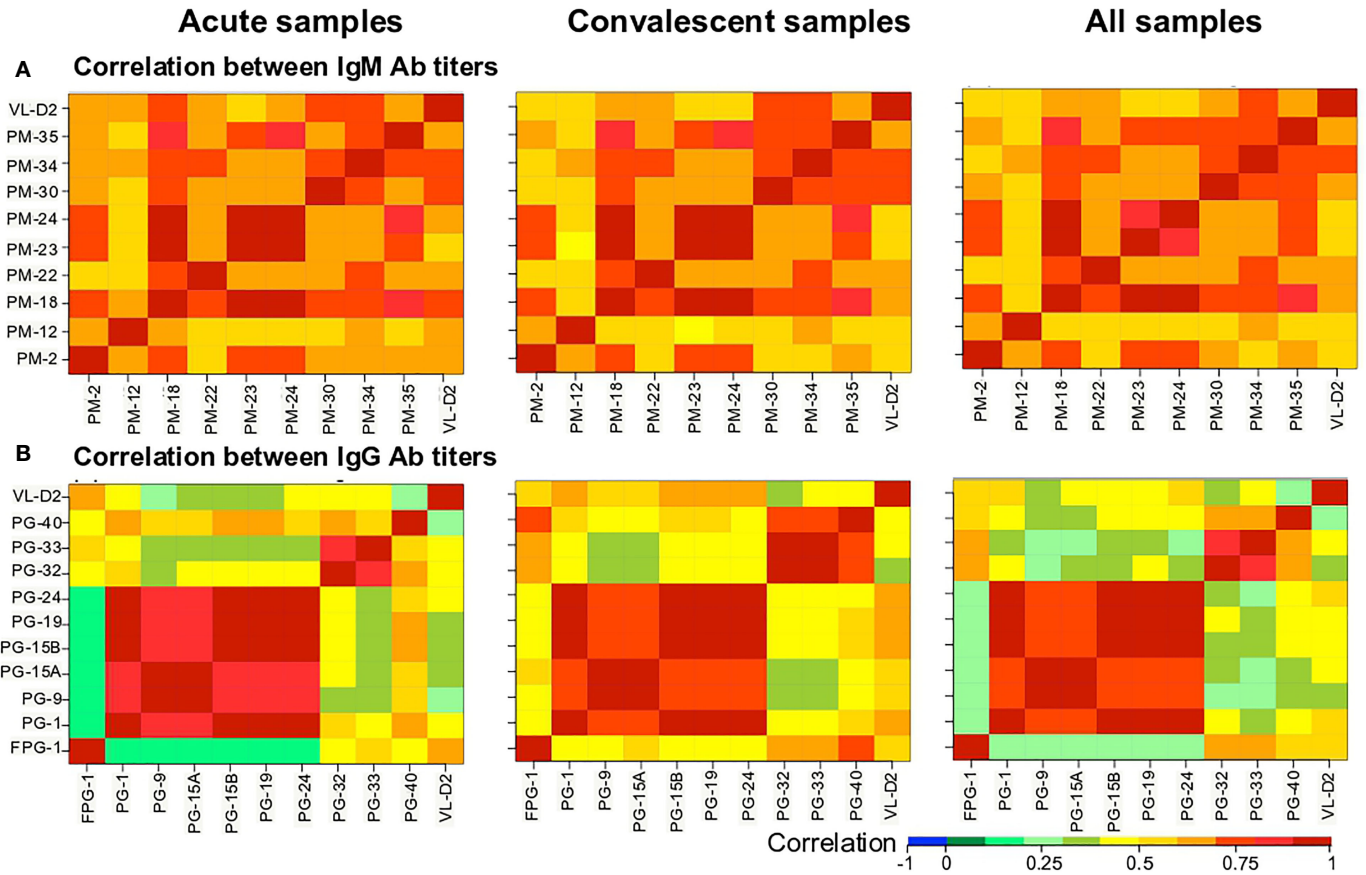
Heatmap of the Spearman’s correlation coefficient between biomarkers. **(A)** IgM and **(B)** IgG Ab responses against the peptides were correlated. Correlation coefficients are indicated by the color scale. Blue indicates a negative correlation; red indicates a positive correlation.

We then evaluated the correlation between the Ab levels against the different biomarkers using the Spearman’s rank correlation test (**Figure 4**). The Ab levels between the nine PM peptides were strongly correlated (**Figure 4A**). In the case of the PG peptides, two groups were observed: (i) a group comprising PG-1, PG-9, PG-15A, PG-15B, PG-19, and PG-24 peptides with a strong correlation between them, that at the same time showed low correlation with the VL-D2, and (ii) a second group of FPG-1, PG-32, PG-33, and PG-40 peptides that showed weak correlation between them, while only FPG-1 showed good correlation with the VL-D2 (**Figure 4B**).

### 3.3 ROC Performance Analysis Combining Multiple Peptides

To determine if the combination of peptides could improve the overall diagnostic performance in terms of sensitivity, specificity and AUC to differentiate DENV infected from non-infected individuals, three RF models were built for each of the PM and PG peptides.

The importance of each biomarker in the outcome variable was ranked by RFs, targeting a minimal specificity of 80%. The variable importance plots for the three RF models are detailed in **Supplementary Figure 3**. A higher Mean Decrease in Accuracy (MDA) indicates higher importance of the variable in the model. The top six peptides ranked in the MDA plots were selected to be included in the training and validation analysis. For PM peptides, PM-22 was ranked first in the three RFM models and PM-2 second for RFM1 and RFM3; while for PG peptides, FPG-1 ranked first for RFG2 and RFG3 and PG-40 had the highest importance in the classification algorithm for the RFG1 model.

**Figure 5:**
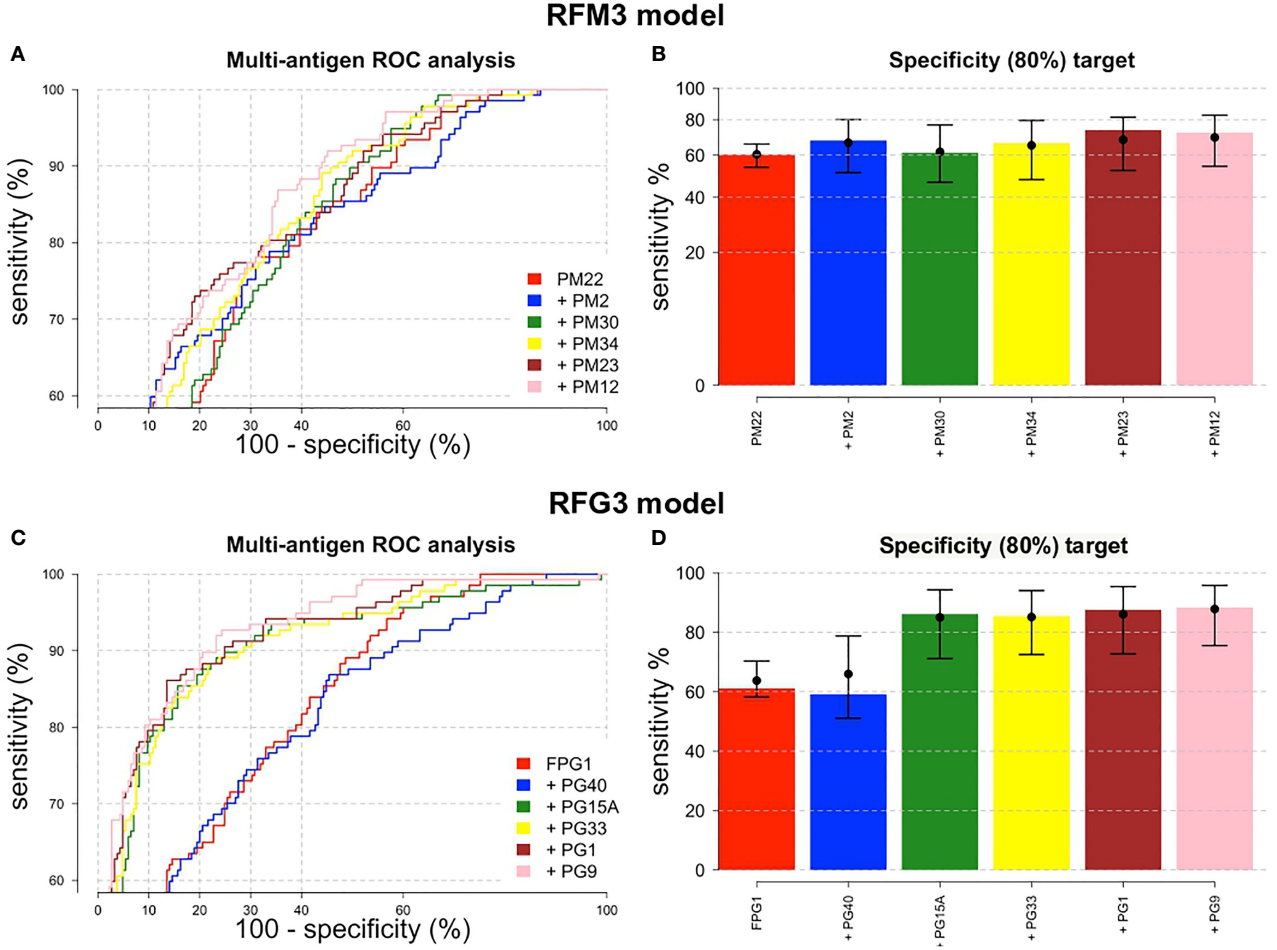
ROC performance analysis combining multiple peptides using a random forest algorithm. ROC curves for **(A)** IgM and **(C)** IgG peptides. The peptides are added sequentially based on their classification accuracy. The axes have been rescaled to better differentiate between high values of sensitivity and specificity. **(B)** For a pre-defined assay specificity of 80%, the assay sensitivity increases with sequentially adding additional peptide biomarkers for detecting IgM **(B)** and IgG **(D)**. Sensitivity was estimated using a random forest classifier. Points and whiskers denote the median and 95% CIs from repeat cross-validation.

The combination of multiple peptides in the RFM and RFG models showed to be superior in terms of sensitivity, specificity and AUC compared to single peptides. For the RFM3 model that included peptides PM-22, PM-2, PM-30, PM-34, PM-23, and PM-12, the sensitivity reached 72.3% (95%CI, 64.2–79.1) (**Figure 5B**). Using the RFM1 and RFM2 models did not improve the diagnostic performance (**Supplementary Table 6**). For the PG peptides, the RFG3 model that included peptides FPG-1, PG-40, PG-15A, PG-33, PG-1, and PG-9 rendered a sensitivity of 88.3% (95%CI, 81.9–92.7) (**Figure 5D**), while higher sensitivities were obtained for the RFG1 model that contained PG-40, PG-15A, FPG-1, PG-19, PG-1, and PG-24 peptides (88.9%, 95%CI, 77.8–94.8) and for the RFG2 model comprising FPG-1, PG-33, PG-9, PG-32, PG-15A, and PG-1 peptides (89.1%, 95%CI, 77–95.3) (**Supplementary Figure 4**). The sensitivity of the RFG3 model was 84.7% (95%CI, 77.7-89.8) and 81% (95%CI, 73.6-86.7) when the targeted specificity was set to a minimum of 85% or 90%, respectively (**Supplementary Figures 4E, F**). The ROC curves for the RFM3 and RFG3 models are shown in **Figures 5A, C**, and for the RFG1 and RFG2 models in **Supplementary Figures 4A, C**. The sensitivity values, targeting a minimal specificity of 80% for the multiple combinations of peptides are shown in **Supplementary Table 6**.

**Figure 6:**
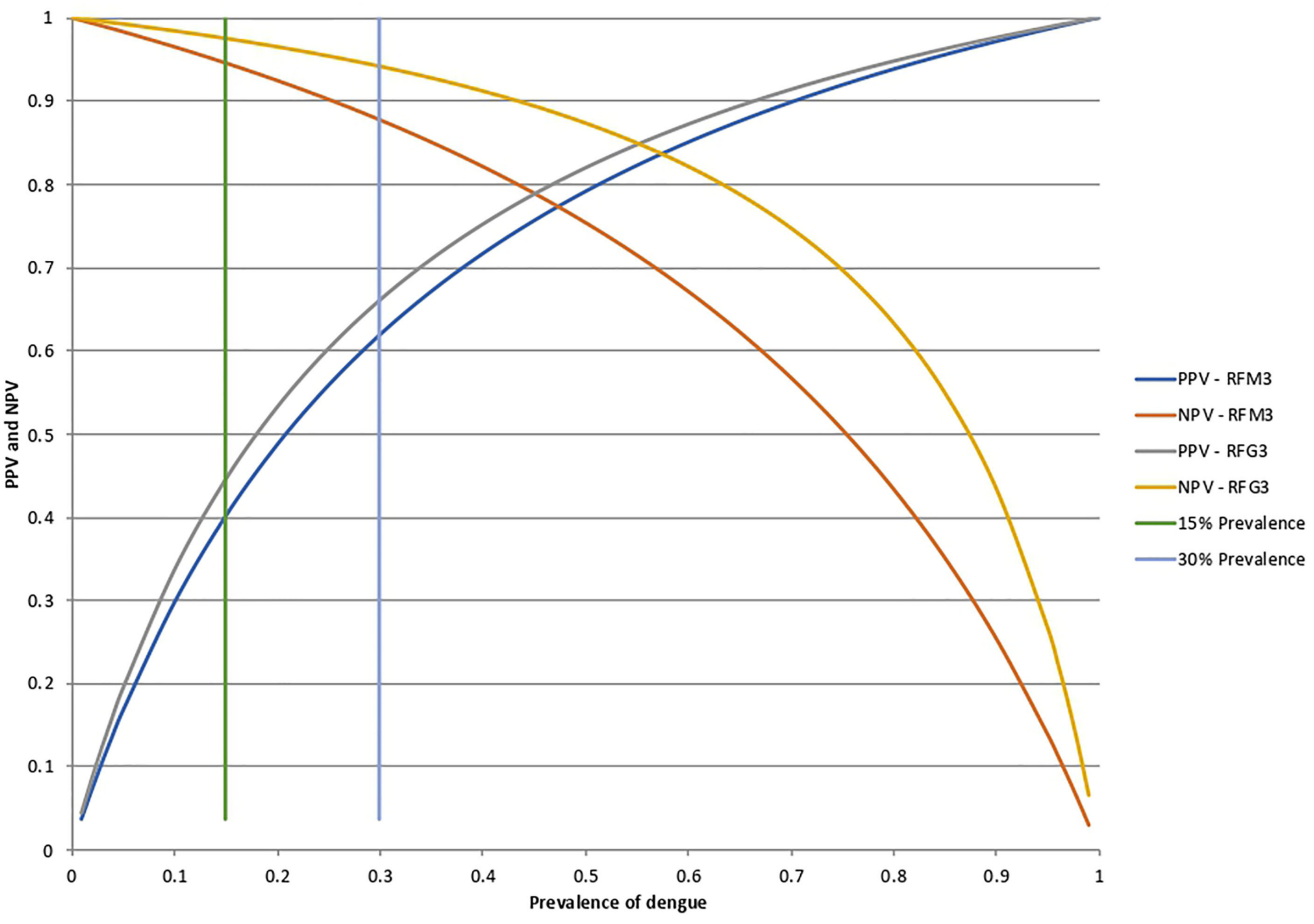
Positive predictive values (PPV) and negative predictive values (NPV) for the RFM3 and RFG3 models. According to the Random Forest analysis for the combination of multiple peptides, the calculated specificity and sensitivity values were fixed at 80% and 74% for the RFM3 model, and 80% and 88% for the RFG3 model, respectively. The RFM3 model included the following peptides: PM-22, PM-2, PM30, PM-34, PM-23, and PM-12. The RFG3 model included the FPG-1, PG-40, PG15A, PG-33, PG-1, and PG-9 peptides. PPV and NPV calculated at a pre-set DENV prevalence of 15% and 30%, which corresponds prevalence which corresponds to the intersection of the horizontal bars with the curves.

The corresponding curves of the relationship between DENV prevalence and the positive (PPV) and negative (NPV) predictive value for the RFM3 and RFG3 models at the calculated sensitivity and specificity are shown in **Figure 6**. For an assumed DENV prevalence of 15% the PPV and NPV will be 40 and 94.6% for the RFM3 model, and 44.4 and 97.5% for the RFG3 model, respectively.

### 3.4 Comparison of MPIA With Commercial Dengue Diagnostic Tests

We compared the results from the Luminex MPIA with two commercial test kits, i.e., DENV ELISA for IgM and IgG (Euroimmun, Lübeck, Germany) and a rapid diagnostic test (RDT) for combined detection of NS1 antigen, IgM and IgG (Standard diagnostics (SD), Yongin-Si, Korea) in a subset of 41 patients from an endemic region and with a confirmed DENV infection (n = 41 acute samples, n = 27 early convalescent samples, n = 14 late convalescent samples). There was a weak but significant positive correlation between the IgG OD ratios from the DENV ELISA and the MFI values for the FPG-1 peptide in the MPIA (r = 0.51, *P* <.001). Also, weak but significant positive correlations were observed between the IgM OD ratios from the DENV ELISA and the MFI values for the peptides PM-18 (r = 0.28, *P* = .0.0099), PM-23 (r = 0.32, *P* = .0037) and PM-24 (r = 0.31, *P* = .0047) in the MPIA (**Supplementary Figure 5A**). We also performed pairwise comparisons between the commercial DENV ELISA and the evaluated peptides. For the PM peptides, we observed that less than 26% of the evaluated samples were positive in both assays and for those peptides that showed significant correlation with the commercial IgM ELISA, acute and early convalescent samples were involved in this correlation (**Supplementary Figure 5B**). While for PG peptides, about 60% of the samples were positive for FPG1 and the IgG ELISA, of which approximately 71% of the samples were convalescent. For the other PG peptides, the percentage of samples that were positive in both assays was less than 45% and the samples were mostly acute phase samples (>75%), except for the PG-32 and PG-33 peptides for which 56 and 62% (respectively) of the samples that were positive in both assays were categorized as convalescent (**Supplementary Figure 5C**).

A more detailed comparison of the MPIA with the DENV ELISA and RDT commercial tests is shown in **Supplementary Figure 6**. The cell plot indicates the classification of each sample as positive or negative for each seromarker used. The classification of the samples as positive or negative when using the RFM 1, 2, and 3 models was the same. Thus, the three RFM models were able to equally identify 32 out of the 41 DENV patients (78%) and they matched in 21 out of the 41 (51%) A-DENV samples and in 12 out of the 27 (44%) E-DENV samples with the DENV IgM ELISA, and in 19 out of 40 A-DENV (47.5%) with the IgM component of the DENV RDT. The RFG1 model identified as positive 36 out of the 41 (88%) DENV acute samples, convalescent sera were available for three out of the five samples classified as negative in the acute sample, but none of them showed antibodies against the combination of peptides included in the RFG1 model. For the RFG3 model, samples seroconverted against peptides multiplexed in this model, for which the positivity percent went from 80% (33/41) in A-DENV samples to 88% (36/41) in E-DENV samples.

## 4 Discussion

Diagnostic testing has a central position in outbreak control. Without diagnostic tests, it is impossible to trace whether people with the disease have infected others, whether the virus persists in survivors, or to investigate the cause of deaths. These objectives can only be accomplished when tests are available with an excellent diagnostic performance. Unfortunately, the current tests available for the detection of Abs against arboviruses in general, and DENV in particular, do not meet these criteria. Specificity represents a major problem given that most of them are based on the use of whole (recombinant) proteins. DENV proteins contain epitopes that are unique to DENV, and also epitopes with high amino acid identity to those present in other flaviviruses (4), therefore in the current context of increasing global circulation of flaviviruses, the use of whole-protein Ags, either natural or recombinant, has intrinsic problems because they can capture cross-reactive Abs.

The use of synthetic peptides as Ags in seroassays present a promising alternative to whole-protein Ags, since immuno-dominant regions with low sequence identity to proteins other than target can be selected, reducing the risk of capturing cross-reactive Abs in the immunoassay. In this regard, extensive analysis has been performed on the characterization of immunodominant epitopes present in the flavivirus structural proteins Capsid, prM and Envelope and the non-structural protein NS1, which are main targets of the humoral immune response (19). Important epitopes present in the other non-structural proteins have also been described to be targeted by Abs (22, 34) and they represent potential Ags to be used in immunoassays. The peptides evaluated in this work span the entire DENV proteome and they were based on their ability to be recognized by Abs present in the serum from DENV infected individuals (22). Among the evaluated peptides, PM-22 from Envelope, PM-30 from NS3, FPG-1 from NS1, PG-15B from NS1, PG-19 from NS2B, PG-24 from NS3 and PG-40 from NS5 were able to classify DENV samples in the positive category and sera from individuals with other flavivirus history in the negative category. Particularly FPG-1 and PM-22 were the most promising biomarkers for application as Ags in new serological tests.

Remarkably, sera from CHIKV-infected returning travelers presented high IgM Abs against some PM peptides evaluated in this study. Since DENV and CHIKV belong to different genera and no Ab cross-reactivity is expected, a possible explanation for this observation is that these CHIKV-infected patients underwent a previous infection with another microorganism or virus different to CHIKV able to induce polyclonal B-cell reactivity B cell activation, producing antibodies that cross-react with the dengue epitopes. This ability to induce poly reactive B-cells has been described for *Plasmodium* spp., for instance it was shown that Malaria-positive sera can react against ZIKV-antigens present in commercial ZIKV-ELISA tests (27) and spike and RBD antigens from SARS-CoV2 (35, 36). We were not able to rule out the possibility that these CHIKV-positive patients had a past exposure to *Plasmodium* parasites. However, these results highlight the need to include sera from more diverse exposure background during the evaluation or validation of serological tests.

Of note, we have used the peptide sequences as they came off the microarray and thus the amino acid sequences have not been optimized to further enhance recognition and binding affinity. Given the individual variability of the Ab-response towards viral Ags (22), also observed in this study against the evaluated peptides, it is evident that a single unique 15-mer peptide is unlikely to offer sufficiently high sensitivity and specificity. Part of the sequence of FPG-1 peptide has been previously reported to be an immunodominant targeted by the immune system following DENV vaccination and natural infection (37), while there are no reports in scientific literature describing the PM-22 peptide sequence located in NS2A as a potential diagnostic antigen.

Interestingly, when comparing the longitudinal Ab responses against FPG-1 in DENV positive patients, it was shown that the responses in the early convalescent phase were higher than in the acute phase, and that this response waned over time (>70 days after symptom onset), which is in agreement with results obtained in our previous study for the Ab response targeting immudominant regions located in the NS1 protein using the microarray platform (22). The low reactivity towards FPG-1 in A-DENV samples from endemic-area patients contrasted with the high IgG titers measured against the antigens present in the commercial ELISA corresponding to virus particles of DENV-2 (data not shown). These results suggest that this biomarker could be useful to diagnose DENV infection based on IgG seroconversion using paired sera in DENV endemic regions where secondary/multiple DENV infections occurs. Despite the fact that NS1 is highly conserved among flaviviruses (38), a good specificity was observed for FPG-1 peptide, since samples from individuals exposed to other flavivirus showed low reactivity.

When the Ab titers from endemic and non-endemic A-DENV sera were compared, we noticed that the magnitude of the Ab response against PG-40 was significantly higher in the endemic group. These results suggest that this peptide could be useful for the differentiation of primary from secondary infection, under the assumption that the rapid rise of IgG titers against PG-40 detected in A-DENV samples from endemic patients corresponded with an anamnestic response from a previous DENV infection. Unfortunately, no documented history of previous DENV infection is available for these samples. PG-40 peptide could also be of special interest as a biomarker for serostatus determination, given that a positive test confirming prior DENV infection is crucial to guide vaccination with Dengvaxia (11). According to a recent review, the current available DENV RDTs are highly specific (100%), but the sensitivity is lower than 41% for the detection of prior infection in endemic samples (12).

We found that hospitalized DENV patients showed significantly higher IgG titers against the PG-33 peptide located in NS3 compare to the titers observed in non-hospitalized individuals, making this peptide a potentially attractive biomarker for disease severity. However, more samples are need to be tested to confirm this finding. No linear continuous immunoreactive peptides from flaviviruses located in the NS3 protein have been previously reported (39).

A fundamental aspect of this work is the use of machine learning classification algorithms to evaluate the diagnostic performance of peptides in order to select the best possible combination that results in the highest possible sensitivity and specificity. For this purpose, the specificity was prioritized over sensitivity given the impact that specificity has on flavivirus diagnosis. These findings revealed that the combination of six different peptides for RFM and RFG models showed an improvement in the sensitivity compared to the observed sensitivity when single peptides were evaluated. Despite that specificity was targeted at minimally 80%, this constitutes a clear progress in respect to the performance of currently available commercial tests for DENV serology.

Our work adds important insights to the growing number of studies that seek for biomarkers for the improved serological diagnosis of flavivirus infections (12, 39, 40). Further modification and subsequent functional analysis of these peptides with a larger number of samples from DENV confirmed cases and from patients with undifferentiated fever is required to further evaluate and prioritize these biomarkers for future DENV test development.

## Data Availability Statement

The original contributions presented in the study are included in the article/**Supplementary Material**. Further inquiries can be directed to the corresponding author.

## Ethics Statement

The studies involving human participants were reviewed and approved by The ethical review boards of the Universidad Peruana Cayetano Heredia, Lima, Peru (Protocol No. 101480), the Institute of Tropical Medicine Antwerp, Belgium (Protocol No. ITG 1304/19) and the University of Antwerp, Belgium (Protocol No. 19/42/477). This study was conducted in compliance with the ethical standards of the latest amended Declaration of Helsinki and of the International Conference Harmonization (ICH) guidelines, plus adhering to local laws and regulations. Written informed consent to participate in this study was provided by the participants’ legal guardian/next of kin.

## Author Contributions

FF-A and KKA wrote the manuscript text. FF-A and KK implemented the analysis. KKA, FF-A, and KK conceived the study. FF-A, XM, and DB processed samples. FF-A analyzed the data. MT, FF-A, XM, and ME wrote study protocols and coordinated sample collection. All authors contributed to the article and approved the submitted version.

## Funding

This work was supported by the Belgian Directorate-general Development Cooperation and Humanitarian Aid (DGD) for the Framework Agreement 4 project (2017–2021), the European Union’s Horizon 2020 research and innovation program, under the ZikaPLAN grant agreement 734584.4, the Research Foundation Flanders (FWO grant number G054820N) (to KKA) and the Flanders Innovation & Entrepreneurship (VLAIO) program for the Innovation mandate [HBC.2018.0327] to KK. FF-A holds a PhD scholarship funded by the DGD. The National Reference Center for Arboviruses of the ITM is partially supported by the Belgian Ministry of Social Affairs through a fund within the Health Insurance System.

## Conflict of Interest

The authors declare that the research was conducted in the absence of any commercial or financial relationships that could be construed as a potential conflict of interest.

The handling editor has declared a past co-authorship with one of the authors, KKA, at the time of review.

## Publisher’s Note

All claims expressed in this article are solely those of the authors and do not necessarily represent those of their affiliated organizations, or those of the publisher, the editors and the reviewers. Any product that may be evaluated in this article, or claim that may be made by its manufacturer, is not guaranteed or endorsed by the publisher.

## Supplementary Material

Supplementary Figure 1 Antibody responses to single peptides among DENV positive samples. Boxplots represent the comparison of the measured Ab response (MFI levels) in DENV positive samples against the detailed peptides between **(A)** endemic and non-endemic samples, **(B)** acute and early convalescent samples, and between early and late convalescent samples, and **(C)** hospitalized versus non-hospitalized samples. Positive DENV samples were confirmed by RT-PCR and/or IgM/IgG seroconversion. Horizontal box boundaries and midline denote sample quartiles. The analysis was performed for all the peptides, but only those showing statistically significant differences between groups are plotted (P <.05).

Supplementary Figure 2 Heat map representing antibody levels against DENV peptides. The heatmap depicts the intensity of the IgM and IgG antibodies measured in MFI. Each column corresponds to one peptide and each row represents a sample. The color intensity indicates the measured MFI values going from low (light yellow) to high (red) levels. DENV, dengue virus; ZIKV, Zika virus; YFV, yellow fever virus; TBEV, Tick borne encephalitis virus; JEV, Japanese encephalitis virus; WNV, West Nile virus; CHIKV, chikungunya virus; HIV, Human immunodeficiency virus; NEG, healthy donors; Conva, convalescent sample.

Supplementary Figure 3 Contribution of each peptide to classification accuracy. Random forest machine learning technique ranked the contribution of each peptide to the classification accuracy using the mean decrease accuracy and the mean decrease gini index. Ranking of **(A)** IgM peptides, and **(B)** IgG peptides. Three different RF models were implemented based on the period since onset of symptoms: acute (≤8 days after symptoms onset), early convalescent (≥10 - ≤70 days after symptoms onset) and all samples (acute + convalescent). The same panel of negative samples were used for the three models.

Supplementary Figure 4 ROC performance analysis combining multiple IgG peptides using a random forest algorithm. ROC curves for IgG peptides in acute RFG1 **(A)** and convalescent RFG2 **(C)** samples. The peptides were added sequentially based on their classification accuracy. The axes have been rescaled to better differentiate between high values of sensitivity and specificity. For a specificity set at 80% **(B**, **D)**, 85% **(E)** and 90% **(F)**, we plotted the respective sensitivity. Sensitivity was estimated using a random forests classifier and peptide biomarkers were added sequentially. Points and whiskers denote the median and 95% CIs from repeat cross-validation.

Supplementary Figure 5 Correlation of antibody titers between individual peptides and the commercial ELISA for dengue. **(A)** Heatmap of the Spearman’s correlation coefficient between the antibody titers against the synthetic peptides and a commercial DENV ELISA. For the commercial kit, the OD (optical density measured at 450 nm wavelength) and the OD ratio (OD values of the sample and the calibrator provided in the kit) were used to calculate the correlation. Correlation coefficients are indicating by the color scale. Blue indicates a negative correlation; red indicates a positive correlation. **(B)** Pairwise correlation between each IgM peptide and the commercial DENV ELISA IgM kit. **(C)** Pairwise correlation between each IgG peptide and the commercial DENV ELISA IgG kit. The antibody response was measured in MFI for the synthetic peptides and OD (Absorbance at 450 nm) for the commercial kit. Each dot represents a sample. Dashed red lines indicate the cut-off values for the commercial kit according the manufacturer instructions (vertical line) and for the peptide based on the ROC curves enforcing a minimum specificity of 80% (horizontal line).

Supplementary Figure 6 Comparison of result outcomes between the random forest models and DENV commercial diagnostic kits. The analysis was performed in a subset of 41 endemic DENV positive samples. **(A)** Cell plot where each column represents a serologic test and each row represents a sample. The analysis for the DENV peptides was done with the combination of peptides based on the random forest analysis. **(B)** Peptide composition of each model for IgM peptides (RFM) and IgG peptides (RFG). **(C)** Differences in classification were assessed by pairwise comparison using Cohen’s kappa and McNemar’s test. The values above the diagonal indicates the kappa coefficient with the 95% CI range for the Cohen’s test while for the MacNemar’s test they represent the Odds ratio. The values below the diagonal in each table corresponds to the p value.
