# Supplementary figures and images for: Peptide Biomarkers for the Diagnosis of Dengue Infection
Source: Front Immunol. 2022 Jan 26;13:793882. doi: 10.3389/fimmu.2022.793882 (PMC8826428; doi:10.3389/fimmu.2022.793882)

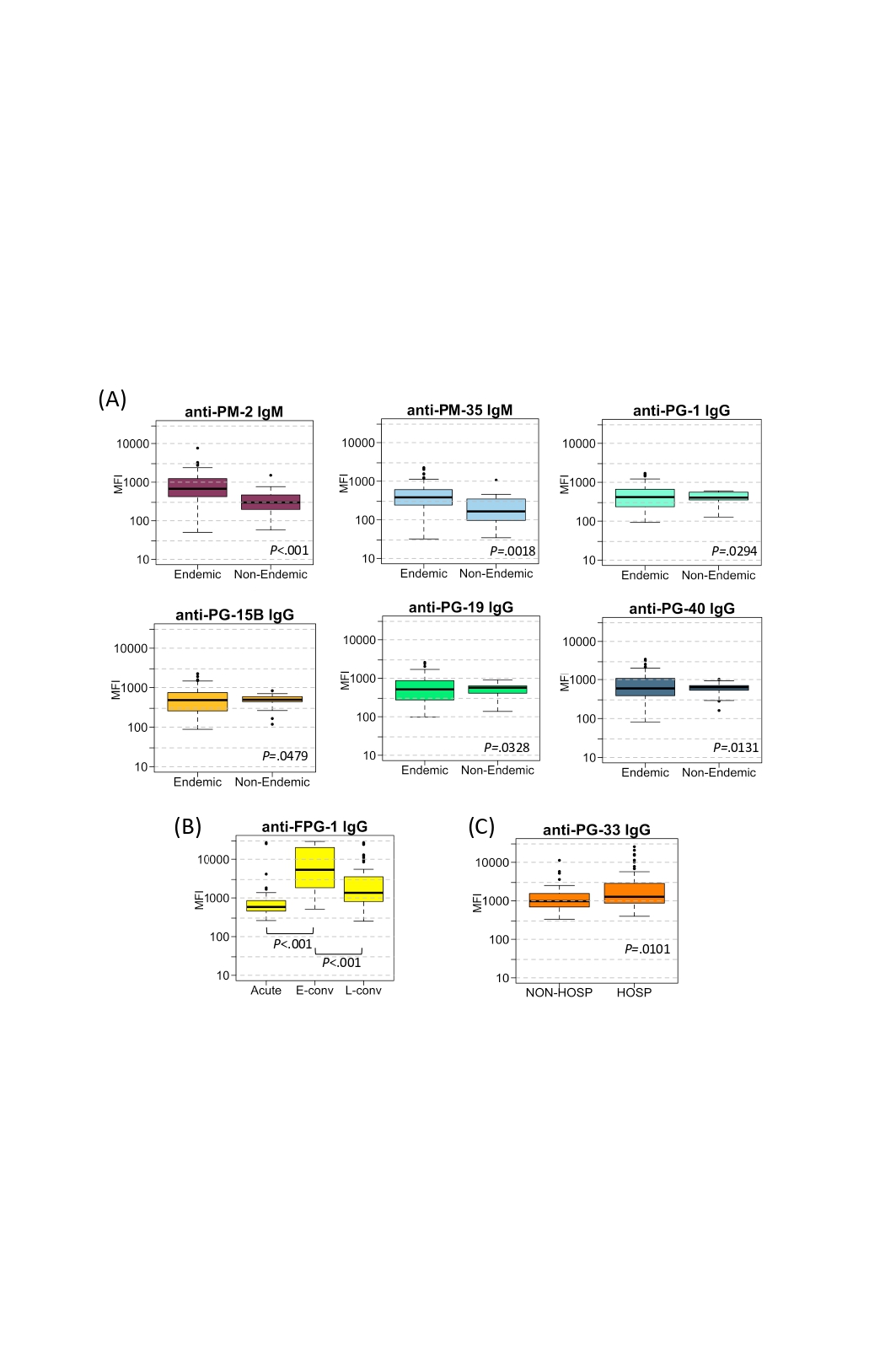

Supplement: Supplementary Figure 1 — Antibody responses to single peptides among DENV positive samples. Boxplots represent the comparison of the measured Ab response (MFI levels) in DENV positive samples against the detailed peptides between (A) endemic and non-endemic samples, (B) acute and early convalescent samples, and between early and late convalescent samples, and (C) hospitalized versus non-hospitalized samples. Positive DENV samples were confirmed by RT-PCR and/or IgM/IgG seroconversion. Horizontal box boundaries and midline denote sample quartiles. The analysis was performed for all the peptides, but only those showing statistically significant differences between groups are plotted (P <.05). [file Image_1.jpeg]

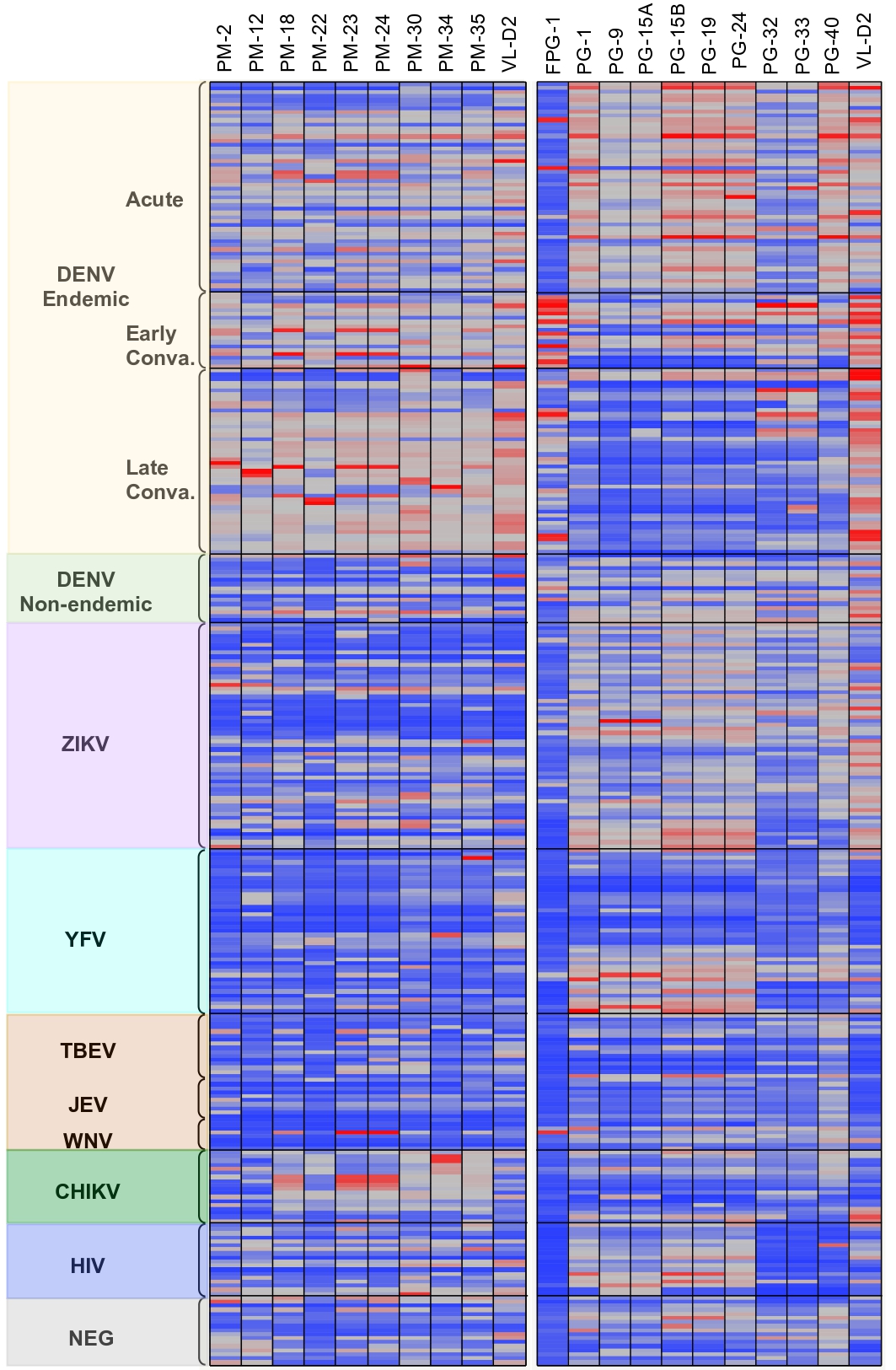

Supplement: Supplementary Figure 2 — Heat map representing antibody levels against DENV peptides. The heatmap depicts the intensity of the IgM and IgG antibodies measured in MFI. Each column corresponds to one peptide and each row represents a sample. The color intensity indicates the measured MFI values going from low (light yellow) to high (red) levels. DENV, dengue virus; ZIKV, Zika virus; YFV, yellow fever virus; TBEV, Tick borne encephalitis virus; JEV, Japanese encephalitis virus; WNV, West Nile virus; CHIKV, chikungunya virus; HIV, Human immunodeficiency virus; NEG, healthy donors; Conva, convalescent sample. [file Image_2.jpeg]

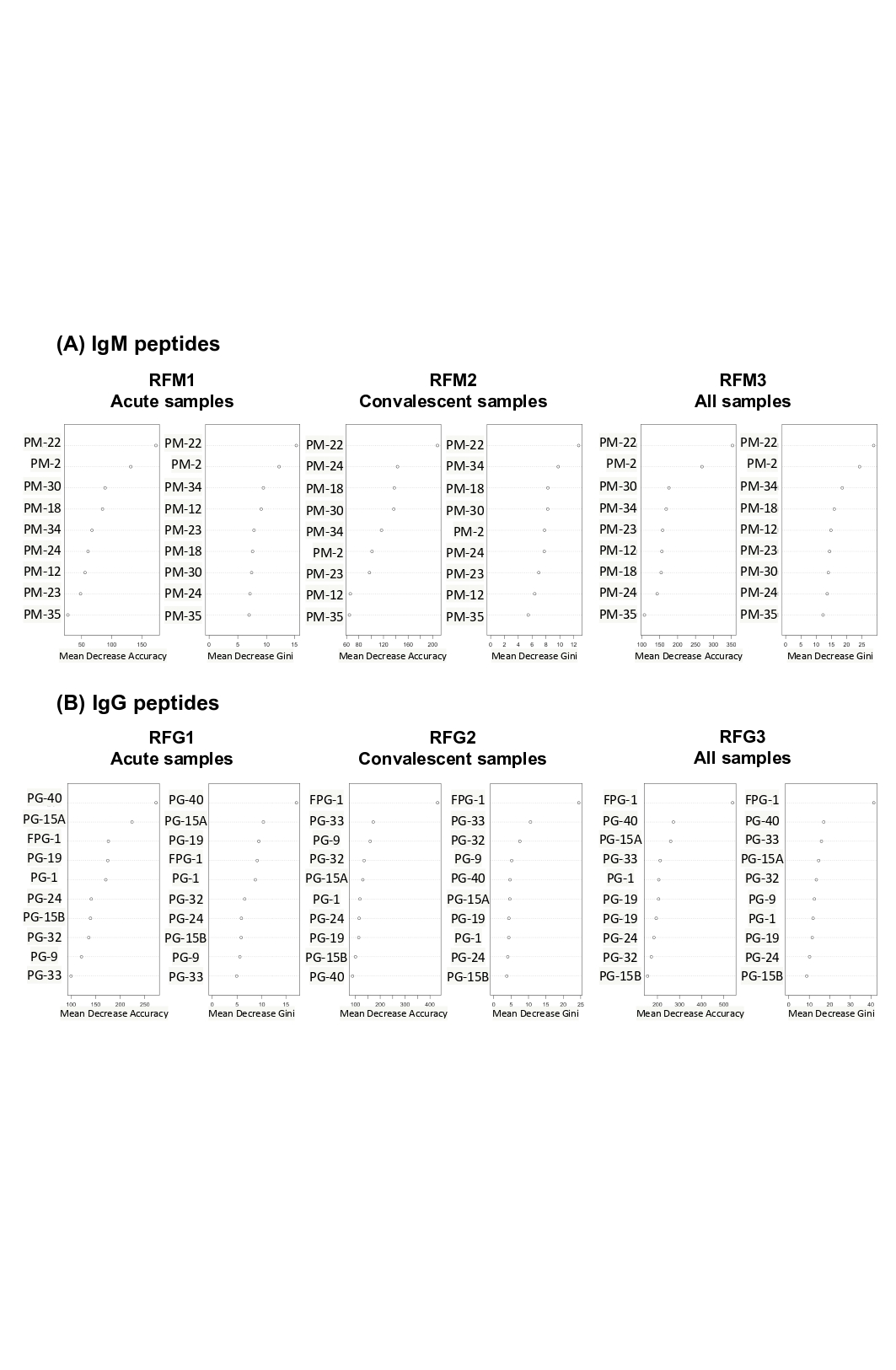

Supplement: Supplementary Figure 3 — Contribution of each peptide to classification accuracy. Random forest machine learning technique ranked the contribution of each peptide to the classification accuracy using the mean decrease accuracy and the mean decrease gini index. Ranking of (A) IgM peptides, and (B) IgG peptides. Three different RF models were implemented based on the period since onset of symptoms: acute (≤8 days after symptoms onset), early convalescent (≥10 - ≤70 days after symptoms onset) and all samples (acute + convalescent). The same panel of negative samples were used for the three models. [file Image_3.jpeg]

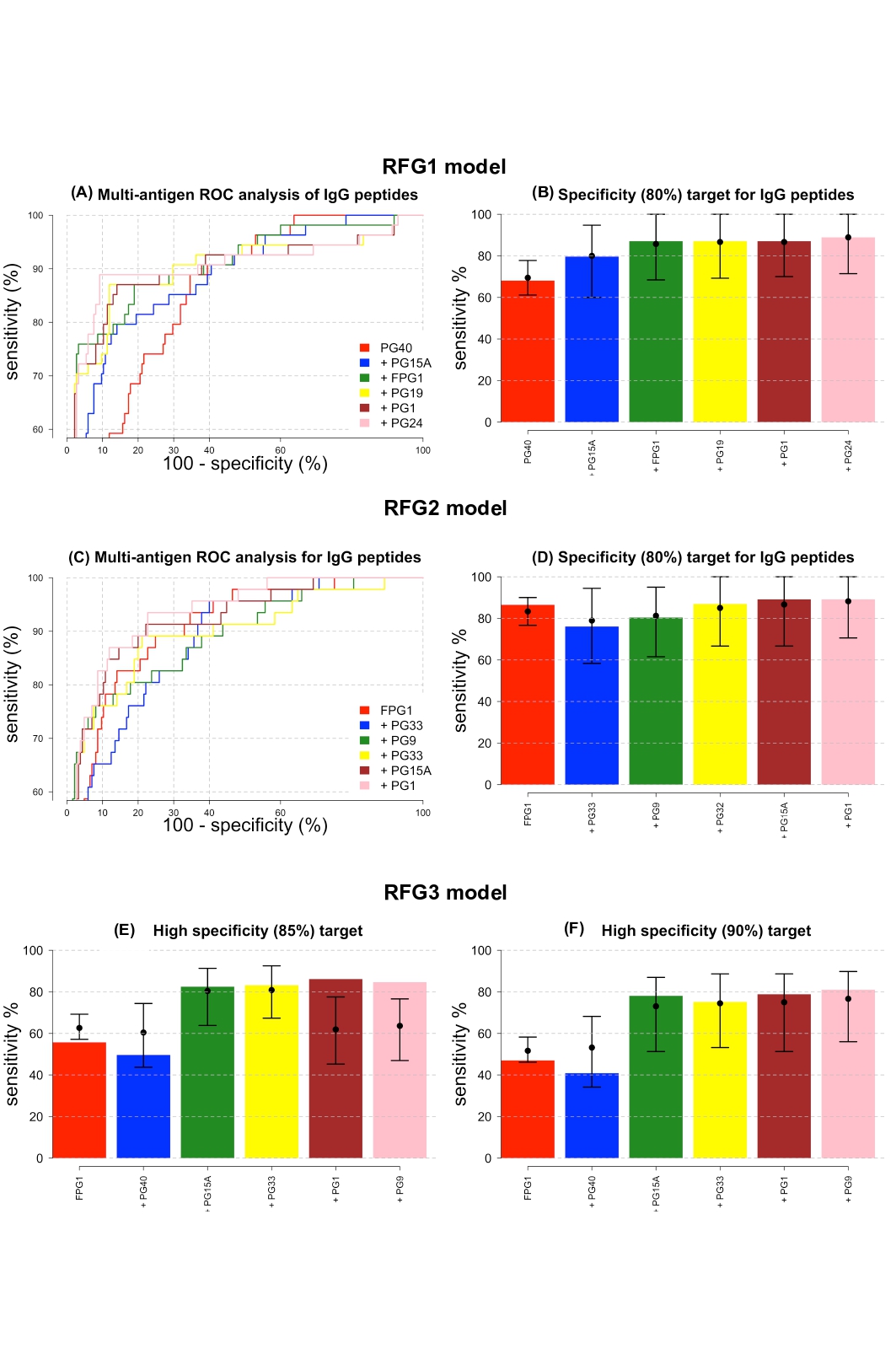

Supplement: Supplementary Figure 4 — ROC performance analysis combining multiple IgG peptides using a random forest algorithm. ROC curves for IgG peptides in acute RFG1 (A) and convalescent RFG2 (C) samples. The peptides were added sequentially based on their classification accuracy. The axes have been rescaled to better differentiate between high values of sensitivity and specificity. For a specificity set at 80% (B, D), 85% (E) and 90% (F), we plotted the respective sensitivity. Sensitivity was estimated using a random forests classifier and peptide biomarkers were added sequentially. Points and whiskers denote the median and 95% CIs from repeat cross-validation. [file Image_4.jpeg]

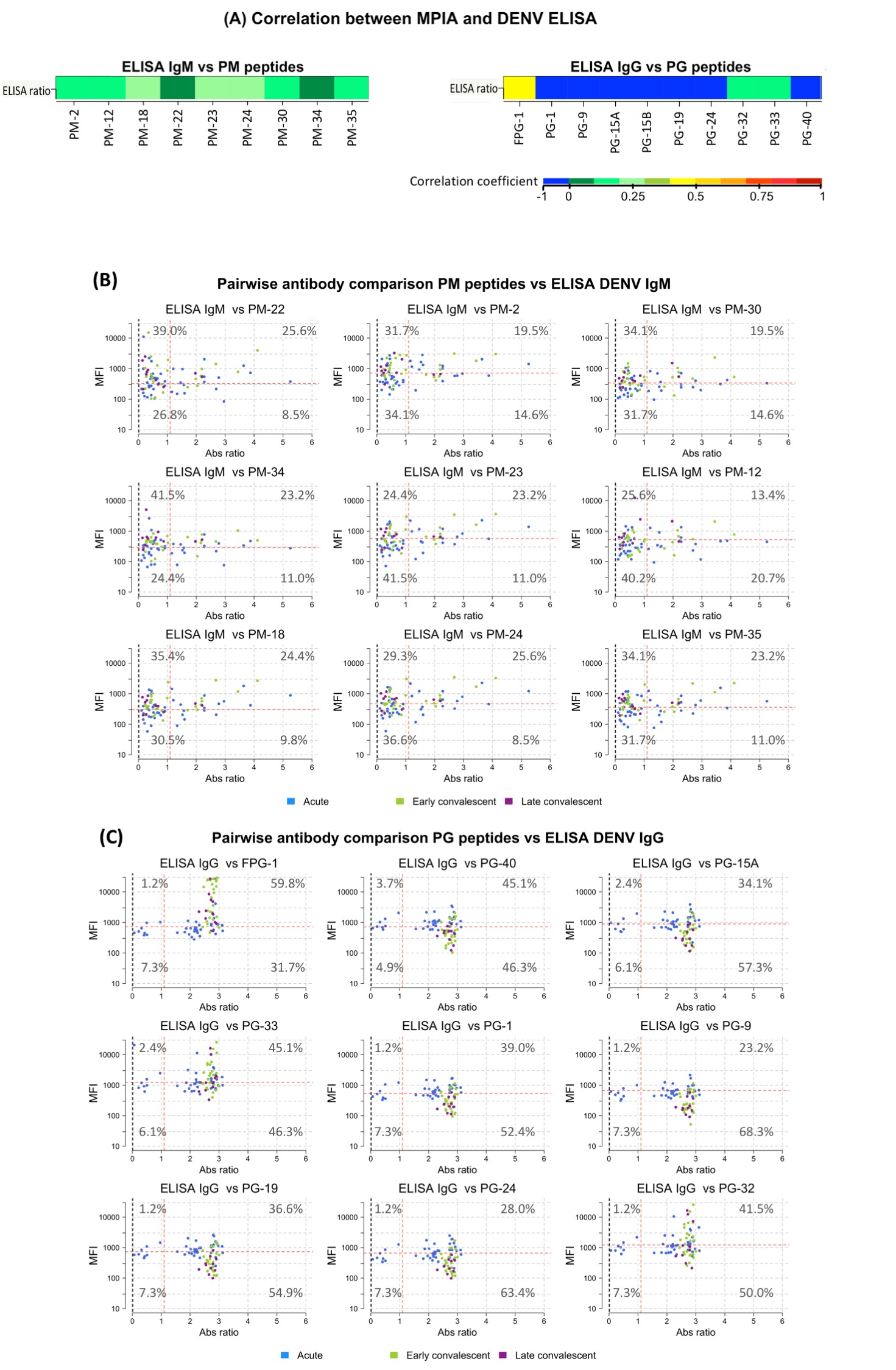

Supplement: Supplementary Figure 5 — Correlation of antibody titers between individual peptides and the commercial ELISA for dengue. (A) Heatmap of the Spearman’s correlation coefficient between the antibody titers against the synthetic peptides and a commercial DENV ELISA. For the commercial kit, the OD (optical density measured at 450 nm wavelength) and the OD ratio (OD values of the sample and the calibrator provided in the kit) were used to calculate the correlation. Correlation coefficients are indicating by the color scale. Blue indicates a negative correlation; red indicates a positive correlation. (B) Pairwise correlation between each IgM peptide and the commercial DENV ELISA IgM kit. (C) Pairwise correlation between each IgG peptide and the commercial DENV ELISA IgG kit. The antibody response was measured in MFI for the synthetic peptides and OD (Absorbance at 450 nm) for the commercial kit. Each dot represents a sample. Dashed red lines indicate the cut-off values for the commercial kit according the manufacturer instructions (vertical line) and for the peptide based on the ROC curves enforcing a minimum specificity of 80% (horizontal line). [file Image_5.jpeg]

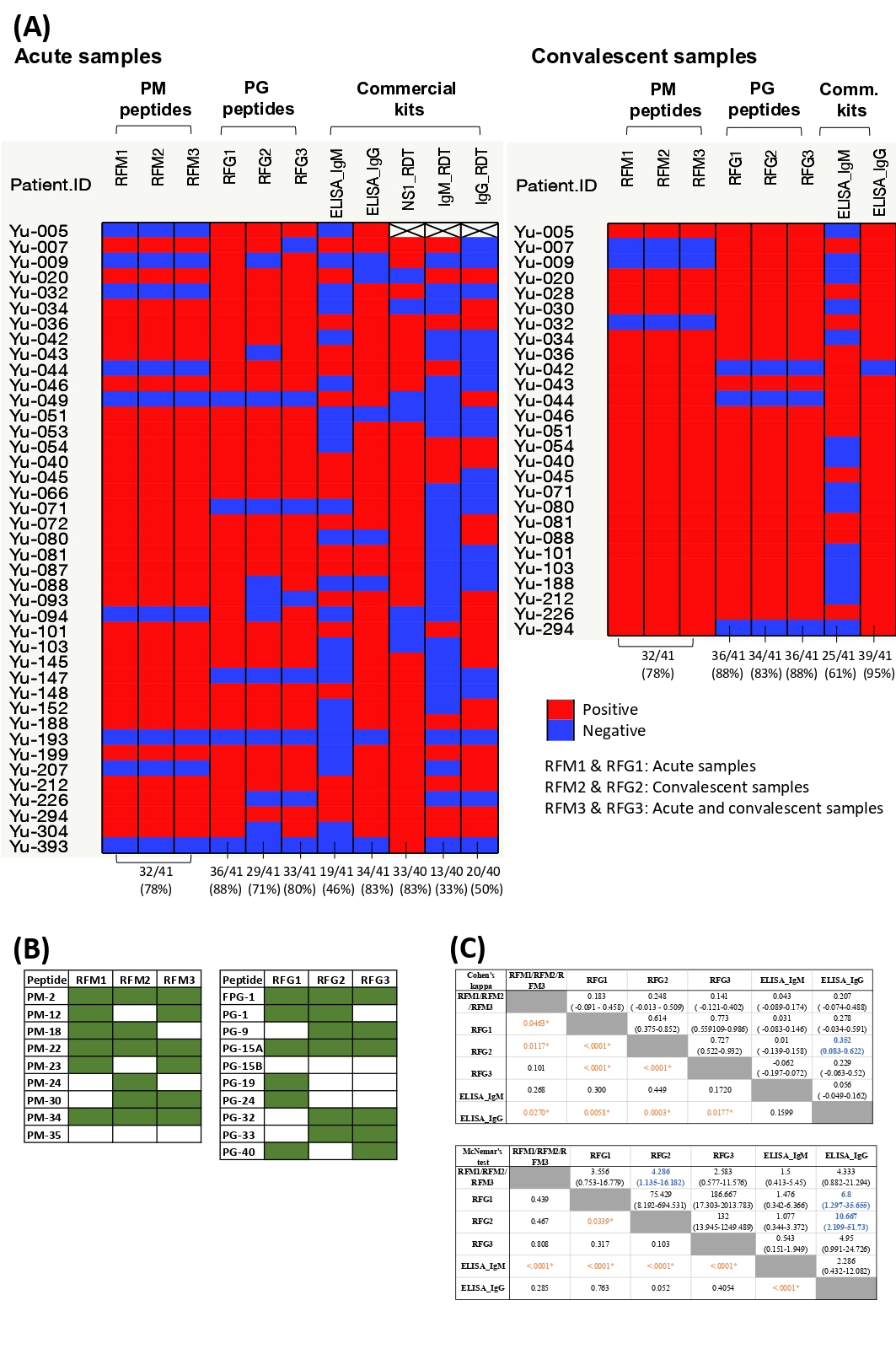

Supplement: Supplementary Figure 6 — Comparison of result outcomes between the random forest models and DENV commercial diagnostic kits. The analysis was performed in a subset of 41 endemic DENV positive samples. (A) Cell plot where each column represents a serologic test and each row represents a sample. The analysis for the DENV peptides was done with the combination of peptides based on the random forest analysis. (B) Peptide composition of each model for IgM peptides (RFM) and IgG peptides (RFG). (C) Differences in classification were assessed by pairwise comparison using Cohen’s kappa and McNemar’s test. The values above the diagonal indicates the kappa coefficient with the 95% CI range for the Cohen’s test while for the MacNemar’s test they represent the Odds ratio. The values below the diagonal in each table corresponds to the p value. [file Image_6.jpeg]
